# Effect of a phthalocyanine mouthrinse after connective tissue graft surgery for root coverage: a randomized split-mouth clinical trial

**DOI:** 10.1007/s10006-026-01614-9

**Published:** 2026-08-06

**Authors:** Rafael Sponchiado Cavallieri, Caique Andrade Santos, Rafaela Stocker Salbego, Carla Andreotti Damante, Adriana Campos Passanesi Santana, Heitor Marques Honório, Leonardo Rigoldi Bonjardim, Mariana Schutzer Ragghianti Zangrando

**Affiliations:** 1 Bauru School of Dentistry, Department of Prosthodontics and Periodontics, University of São Paulo, Vila Nova Cidade Universitária, Al. Dr. Octávio Pinheiro Brisolla, 9-75, Bauru, São Paulo, 17012-901 Brazil; 2 Bauru School of Dentistry, Departament of Biological Sciences, University of São Paulo, Vila Nova Cidade Universitária, Al. Dr. Octávio Pinheiro Brisolla, 9-75, Bauru, São Paulo 17012-901 Brazil; 3 Bauru School of Dentistry, Department of Pediatric Dentistry, Orthodontics and Public Health, University of São Paulo, Al. Dr. Octávio Pinheiro Brisolla, 9-75, Vila Nova Cidade Universitária, Bauru, São Paulo 17012-901 Brazil

**Keywords:** Gingival recession, Subepithelial connective tissue graft, Chlorhexidine, Phthalocyanine, Patient Reported Outcome Measures, Randomized clinical trial

## Abstract

**Objectives:**

To evaluate effects of self-activating phthalocyanine mouthrinse on recipient sites of subepithelial connective tissue graft (SCTG), considering clinical outcomes, professional and patient assessments and quantitative somatosensory changes.

**Materials and methods:**

This randomized controlled, crossover, split-mouth clinical trial included 20 patients treated with SCTG plus coronally advanced flap (CAF). Recipient sites received 0.12% iron phthalocyanine mouthrinse (test group-PHY) and 0.12% chlorhexidine (control group-CHX) during 14 postoperative days. The primary outcome was percentage of root coverage (%RC) at 6 months. Secondary outcomes included clinical parameters (probing depth, PD; recession depth and width, RD, RW; percentage of complete root coverage (CRC); clinical attachment level, CAL; keratinized tissue thickness and width, KTT, KTW), professional- and patient-reported outcomes, mechanical detection threshold (MDT) and mechanical pain threshold (MPT).

**Results:**

PHY and CHX groups showed significant improvements in clinical parameters, including reduced RD and RW, CAL gain, and increased KTW and KTT (*p* < 0.05). %RC was similar between groups, at approximately 80%. Professional- and patient-reported outcomes, including dentin hypersensitivity and esthetic perception, as well as MPT and MDT, showed no significant differences between groups. Postoperative symptoms decreased progressively, and oral health-related quality of life remained unchanged after 6 months.

**Conclusions:**

Postoperative use of phthalocyanine after CAF + SCTG resulted in clinical outcomes comparable to chlorhexidine, including similar root coverage, with no differences in professional-, patient-centered outcomes, somatosensory profile, or oral health-related quality of life.

**Clinical Relevance:**

Iron phthalocyanine may represent a promising alternative for postoperative care in periodontal plastic surgeries.

## Introduction

The exposure of the root surface resulting from the apical migration of the gingival margin, known as gingival recession (GR), is a condition commonly observed in clinical practice and may negatively impact both function and esthetics [1–3]. With a multifactorial etiology, GR can be associated with mechanical trauma from toothbrushing, thin phenotype and orthodontic movements [4–7]. When multiple teeth are affected, clinical challenges increase, requiring more comprehensive treatments and well-planned, effective surgical approaches [8–11].

Among the surgical techniques available for root coverage, the use of a subepithelial connective tissue graft (SCTG) associated with a coronally advanced flap (CAF) presents the most robust outcomes in the literature [12–15]. However, the need to harvest the graft from the palate is associated with postoperative morbidity, discomfort, and risk of complications such as bleeding, pain, and temporary sensory alterations [16–18]. In addition, the recipient site also requires specific care, including biofilm control, protection against mechanical trauma, and flap stability [19].

In this context, chlorhexidine remains the gold-standard plaque control agent during the early postoperative period because of its well-established antimicrobial effectiveness. However, its use has been associated with adverse effects, including tooth staining, taste alteration, and cytotoxic effects on fibroblasts involved in periodontal wound healing [20–24]. These limitations have encouraged the search for safer and more biocompatible alternatives. Among the emerging options, iron phthalocyanine derivatives have attracted increasing interest because of their antimicrobial potential and favorable biocompatibility profile. Iron phthalocyanine is a self-activating compound capable of generating reactive oxygen species in the presence of molecular oxygen, promoting antimicrobial activity without the need for external light activation [25, 26]. A preclinical study demonstrated lower cytotoxicity of iron phthalocyanine compared with chlorhexidine in gingival fibroblasts, suggesting its potential to better preserve cellular events involved in periodontal wound healing [26]. Furthermore, preliminary clinical evidence, including a recent case series evaluating palatal wound healing after connective tissue graft harvesting, has suggested favorable tissue repair associated with iron phthalocyanine [27].

In addition to clinical aspects involved in the treatment of GR, the patient perspective has become increasingly relevant. Somatosensory evaluation of donor and recipient sites has emerged as a useful tool for identifying possible sensory alterations resulting from surgical manipulation. Although the literature is still limited, studies using qualitative somatosensory analyses [28, 29] indicated that such alterations are generally transient and result in non-significant functional impairment. Nevertheless, postoperative sensory monitoring remains relevant both for surgical planning and for appropriate management of patient-reported complaints.

Beyond sensory alterations, other subjective postoperative manifestations such as pain, functional discomfort, and limitations in daily activities have gained prominence as relevant parameters for evaluating therapeutic outcomes. These patient-reported outcomes provide complementary information to traditional clinical data and contribute to a more comprehensive understanding of the impact of treatment on the individual’s quality of life. To assess these perceptions, validated instruments such as visual analog scales (VAS) and questionnaires like the Oral Health Impact Profile (OHIP-14) have been widely used. Available evidence indicates that discomfort following periodontal surgeries is generally mild and transient, although it may vary according to the type and duration of the intervention. The systematic incorporation of these indicators not only enhances the assessment of clinical outcomes but also supports more personalized and patient-centered treatment approaches [16, 30, 31].

Despite promising in vitro evidence demonstrating antimicrobial activity and lower cytotoxicity [25, 26], as well as encouraging preliminary clinical findings [27], clinical evidence regarding the use of iron phthalocyanine during periodontal plastic surgery remains limited. Moreover, no randomized controlled clinical trial has comprehensively evaluated its effects on clinical outcomes, patient-reported outcomes, and quantitative somatosensory responses following SCTG associated with CAF.

Therefore, the present randomized split-mouth controlled clinical trial aimed to evaluate clinical, quantitative somatosensory, and patient-centered outcomes in the treatment of multiple GR with SCTG plus CAF, comparing the effectiveness of two postoperative antiseptic protocols: iron phthalocyanine and chlorhexidine gluconate. The null hypothesis (H₀) of this study was that the use of the two postoperative protocols would not result in statistically significant differences in root coverage outcomes.

## Materials and methods

This randomized, controlled, crossover, split-mouth clinical trial was conducted at the Periodontics clinic of Bauru School of Dentistry, University of São Paulo (FOB-USP). The study protocol was approved by the local Ethics Committee (CAAE: 19692019.1.0000.5417), and all participants provided written informed consent. The trial was registered at the Brazilian Registry of Clinical Trials (ReBEC – RBR-93ccq38) and conducted in accordance with the CONSORT guidelines.

Inclusion criteria comprised the presence of bilateral multiple gingival recessions classified as RT1 or RT2, with at least one defect ≥ 2 mm in maxillary canines, premolars, or molars. In these areas, if there were root abrasion/restored root surfaces, only those with steps ≤ 1 mm after removal of the restoration, were included. Exclusion criteria included smoking, pregnancy or lactation, tooth mobility, history of periodontal disease, previous root coverage procedures, use of medications affecting healing, and poor oral hygiene (plaque and bleeding scores > 20%). All patients received initial periodontal therapy prior to surgery.

Sites were randomly allocated to two groups: CHX (control group-chlorhexidine 0.12%) and PHY (test group- iron phthalocyanine 0.12%). First, participants were allocated into 1:1 blocks to balance the order of protocol application (CHX and PHY) and reduce bias in subjective outcomes. Within each block, simple randomization was applied to determine the side (right or left) for the application of PHY or CHX. Allocation was concealed using opaque envelopes, which were opened only at the time of surgery. The examiner responsible for outcome assessment was blinded to group allocation. Participants were also blinded to the assigned mouthrinse, as both products were provided in identical bottles with the same instructions for use. Randomization was performed using the Random Sequence Generator software (www.random.org) and Microsoft Excel.

Patients were instructed to rinse with the assigned mouthrinse twice daily for 14 days and to refrain from mechanical plaque control at the surgical site, while maintaining routine oral hygiene in the remaining areas. A washout period of 45 days was established between surgical interventions. Previous studies have adopted washout periods of at least 15 days for split-mouth designs [32–34]. In the present study, a longer interval was chosen to ensure complete postoperative recovery and maximize patient comfort. Compliance with the prescribed mouthrinse regimen and adverse events were monitored using a standardized postoperative diary, which was reviewed by the investigator at the follow-up appointment [31].

**Fig. 1:**
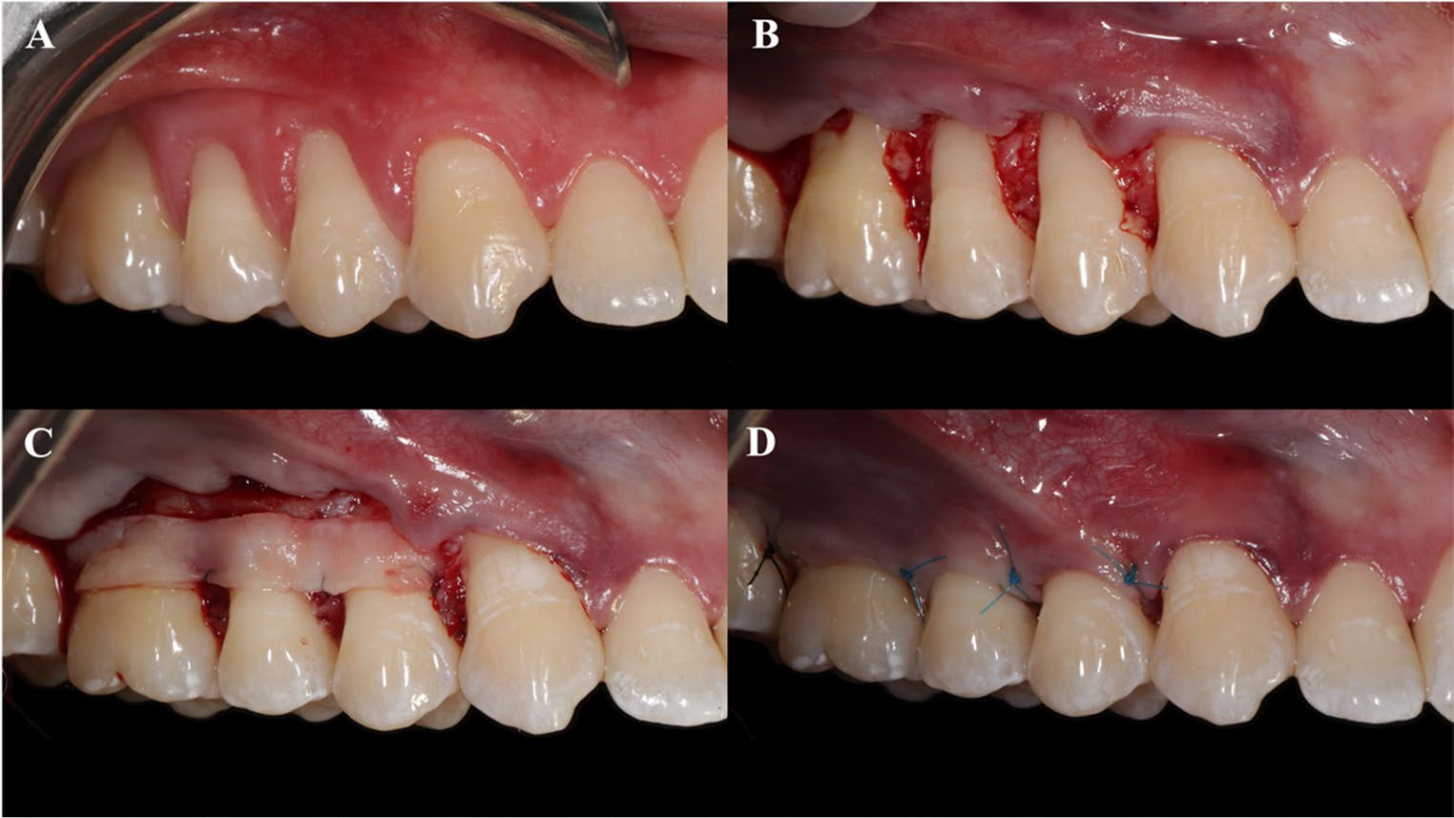
Representative surgical clinical sequence of the coronally advanced flap (CAF) associated with subepithelial connective tissue graft (SCTG). (**A**) Baseline clinical gingival recessions. (**B**) Flap preparation. (**C**) Positioning of SCTG at the cementoenamel junction. (**D**) Final flap positioning and suturing

All surgeries were performed by a single calibrated operator (RSC) under 3.5× magnification. GR were treated using SCTG plus CAF [35]. SCTG were harvested from the palate using a de-epithelialization technique [36, 37] and standardized to a thickness of approximately 1 mm. SCTG was positioned at the cementoenamel junction and stabilized with 5 − 0 nylon sutures, while the flap was coronally advanced and stabilized with sling sutures (Fig. 1). The pharmacological protocol followed the guidelines established by the Periodontics Department of FOB-USP and consisted of a preoperative dose of 8 mg dexamethasone and 100 mg nimesulide twice daily for 4 days. In case of pain, 750 mg paracetamol was recommended as rescue medication. Patients also received written instructions regarding postoperative home care for the first 14 days.

The primary outcome of the present study was the percentage of root coverage (%RC) at the 6-month follow-up. Secondary outcomes included clinical periodontal parameters (Probing depth (PD), clinical attachment level (CAL), plaque index (PI), bleeding on probing (BOP), recession depth (RD), recession width (RW), percentage of complete root coverage (CRC), keratinized tissue width (KTW), and keratinized tissue thickness (KTT)), quantitative somatosensory outcomes (MDT and MPT), professional- and patient -reported outcome measures (PROMs).

Clinical parameters were assessed at baseline and at 6 months using a UNC-15 periodontal probe (Hu-Friedy, Chicago, IL, USA). The blinded examiner responsible for data collection (CAS) was previously calibrated, repeating RD measurements in a set of patients on two separate occasions prior to the study. Intra-examiner reliability was assessed using the intraclass correlation coefficient, demonstrating excellent agreement (ICC = 0.93).

PD, CAL, PI, BOP [38], RD, RW, KTW and KTT [39, 40] were recorded. KTW was defined as the distance from the gingival margin to the mucogingival junction measured at the mid-buccal aspect of the tooth. KTT was assessed by the transgingival probing method at a point 1 mm apical to the gingival margin in the mid-buccal aspect of the tooth with an endodontic spreader and silicone stop, and the penetration depth was measured with a digital caliper.

Dentin hypersensitivity was assessed using an air stimulus and recorded by both the patient (DHp) and the examiner (DHe), using a visual analogue scale (VAS) [41] and the Schiff sensitivity scale [42], respectively. Patient-reported esthetic evaluation (Ep) was also assessed using a VAS score.

Root coverage Esthetic Score (RES) [43], %RC and CRC were evaluated at 6 months. The %RC was calculated from the mean RD of the treated defects at baseline and at 6 months using the following formula: %RC = (mean RD_baseline – mean RD_6 months) / mean RD_baseline × 100 [13, 44].

**Fig. 2:**
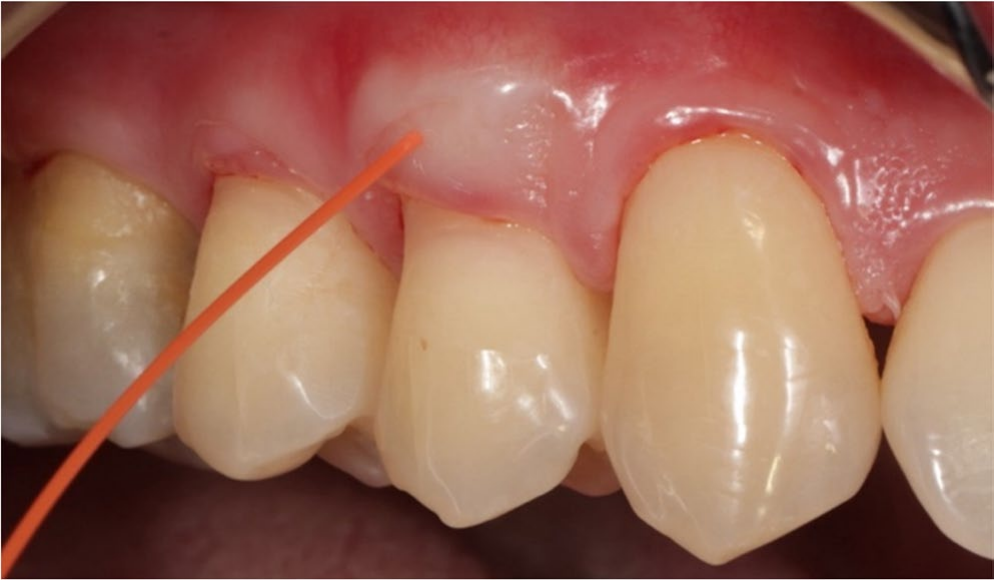
Application of Von Frey filaments for somatosensory assessment of the graft recipient site. Mechanical detection threshold (MDT) and mechanical pain threshold (MPT) were evaluated using calibrated nylon monofilaments applied at standardized points in the recipient area

The quantitative evaluation of somatosensory profile of the recipient sites was performed using Mechanical Detection Threshold (MDT) and Mechanical Pain Threshold (MPT) tests with Von Frey filaments, at baseline and at 2 and 6 months after surgery (Fig. 2), according to established quantitative sensory testing protocols [45–48].

**Fig. 3:**
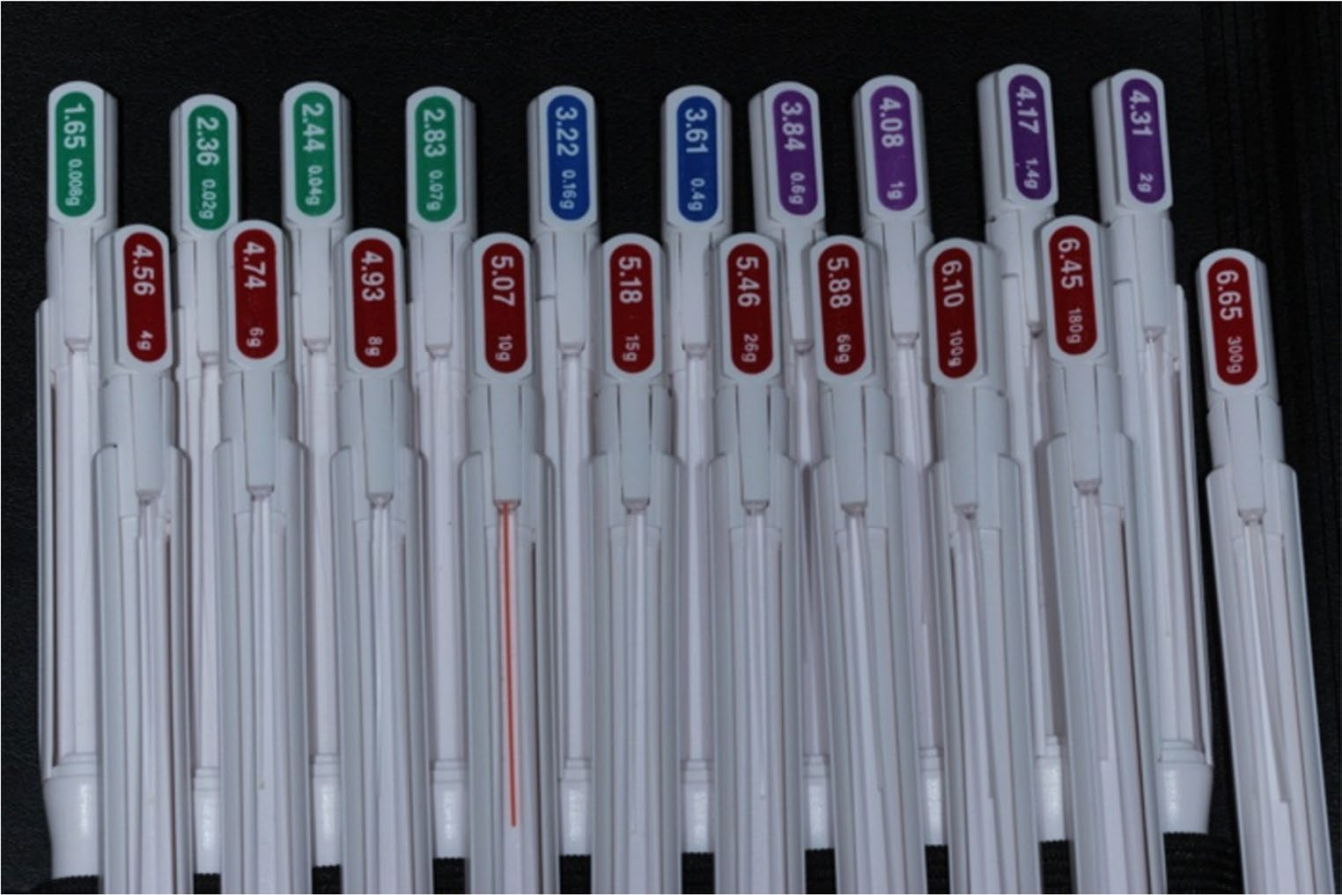
Von Frey filaments for somatosensory assessment of the graft recipient site

For MDT assessment, Semmes–Weinstein nylon monofilaments (0.008–300 g/mm²) (Fig. 3) were used to determine the lowest tactile stimulus perceived [47]. Participants kept their eyes closed and reported when a “light touch” was perceived. Stimuli were applied at 1.0–1.5 mm apical to the gingival margin in the graft recipient area. Filaments were applied in ascending order until perception (positive response), followed by descending application until no perception (negative response). This ascending–descending sequence was repeated until five positive and five negative responses were obtained, and the threshold was calculated as the geometric mean [45, 46, 48]. MPT was assessed using the same protocol, with participants indicating when the stimulus was perceived as painful (e.g., “prickling” or “stabbing”). Threshold calculation followed the same criteria as MDT [45].

The sample size calculation was based on the primary outcome (%RC); therefore, the study may not have been specifically powered to detect differences in secondary outcomes. Considering a split-mouth design with paired comparisons, an expected standard deviation of 20% for %RC [49], an equivalence margin of 15%, a statistical power of 80%, and a significance level of 5%, a minimum of 18 patients was required. To compensate for possible dropouts, 20 patients presenting bilateral defects were included in the study. As multiple gingival recession defects were treated on each side, the mean value of all defects within each treated side was calculated for each participant and for clinical variables. These side-level mean values were considered the unit of analysis, accounting for the clustering effect of multiple defects within the same participant. Statistical analyses were performed using Statistica 10.0 (StatSoft Inc., Tulsa, USA). A two-way repeated measures ANOVA was used to evaluate the effects of group, time, and their interaction. Multiple comparisons were performed using the Tukey test. Normality was assessed using the Shapiro–Wilk test, and normally distributed variables (%RC, KTT, and RES) were additionally analyzed using paired t-tests. The level of significance was set at 5%.

## Results

**Fig. 4:**
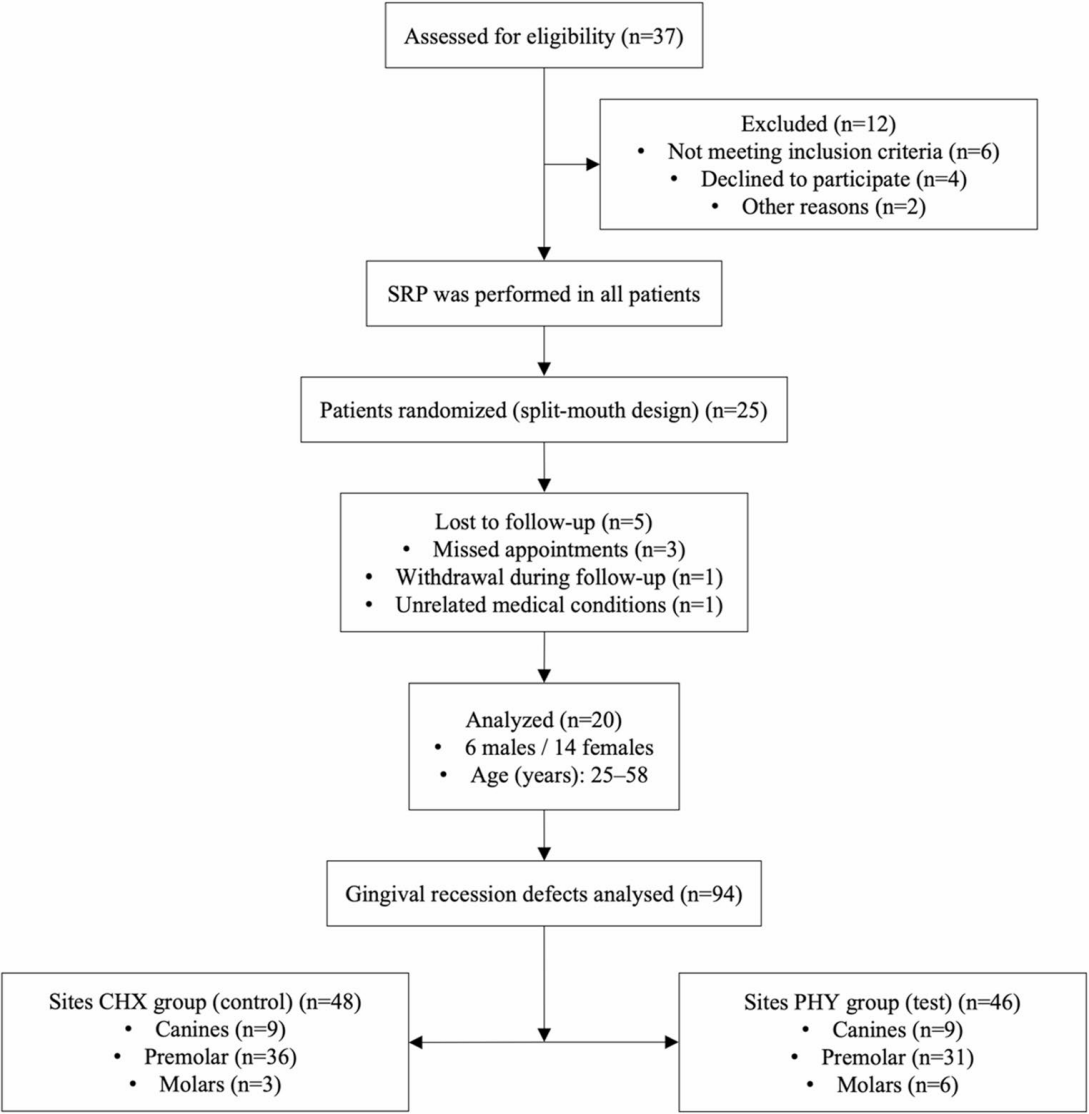
Flow diagram of participant selection, randomization, follow-up, and analysis

Thirty-seven individuals were initially enrolled. After dropouts and exclusions during follow-up, 20 participants (6 males and 14 females; 25–58 years) completed the study (Fig. 4).

A total of 94 gingival recessions were treated using SCTG plus CAF, with 46 defects in PHY group (9 canines, 6 molars, and 31 premolars) and 48 in CHX group (9 canines, 3 molars, and 36 premolars). Baseline demographic and clinical characteristics of the participants and treated recession defects are summarized in Table 1.

**Table 1:** Baseline demographic characteristics of the participants and clinical characteristics of the treated gingival recession defects

Variable	Total	CHX (Control)	PHY (Test)
Participants (n)	20	–	–
Age (years), mean ± SD	44.2 ± 8.2	–	–
Age range (years)	44 (25–58)	–	–
Sex			
Female	14 (70.0%)	–	–
Male	6 (30.0%)	–	–
Gingival recession defects (n)	94	48 (51.1%)	46 (48.9%)
Premolars	67 (71.3%)	36 (75.0%)	31 (67.4%)
Molars	9 (9.6%)	3 (6.3%)	6 (13.0%)
Canines	18 (19.1%)	9 (18.8%)	9 (19.6%)
Recession type			
RT1	57 (60.6%)	32 (66.7%)	25 (54.3%)
RT2	37 (39.4%)	16 (33.3%)	21 (45.7%)

Values are presented as mean ± standard deviation (SD), median (min–max), or number of defects (percentage), as appropriate

*CHX* chlorhexidine (control group), *PHY* phthalocyanine (test group), *RT* recession type according to the Cairo classification

In the intergroup analysis (Table 2), a statistically significant difference was observed only for PI (*p* < 0.05), with higher values in PHY group at 6 months compared with CHX. No significant differences were observed between groups for the other clinical parameters.

In the intragroup analysis (Table 2) over time, both groups demonstrated significant CAL gain and reduction in BOP, with no significant changes in PD. RD and RW were significantly reduced, while KTW KTT significantly increased over time in both groups. No significant differences between groups were observed for %RC at 6 months. Regarding this parameter, the split-mouth comparison showed no statistically or clinically significant difference between treatments. The mean difference between test and control sites was − 0.07 (test − control), with a 95% confidence interval ranging from − 0.19 to 0.05. The effect size was small (Cohen’s dz = − 0.22), indicating a negligible difference between interventions. These findings indicated comparable clinical performance between CHX and PHY mouthrinses in terms of root coverage outcomes at 6 months.

In addition to the %RC, CRC was assessed as a complementary clinical outcome. At the defect level, CRC was achieved in 13 out of 20 sites (65.0%) in the control group and in 9 out of 20 sites (45.0%) in the test group. Considering the patient level in this split-mouth design, CRC was achieved in both treated sites in 9 out of 20 patients (45.0%).

DHp did not show significant changes during the follow-up period, whereas DHe demonstrated a significant reduction. Ep improved significantly throughout the study period (*p* < 0.05). No intergroup differences were observed for RES at 6 months. (Table 2).

**Table 2:** Clinical parameters at baseline and 6 months for CHX and PHY groups

Parameter	Time	CHX	PHY	*p*-value (group)	*p*-value (time)	*p*-value (interaction)
PD (mm)	Baseline	1.61 ± 0.31	1.70 ± 0.51			
	6 months	1.55 ± 0.53	1.56 ± 0.46	0.62	0.36	0.71
RD (mm)	Baseline	2.04 ± 0.77	1.90 ± 0.75			
	6 months	0.35 ± 0.57	0.46 ± 0.50	0.92	< 0.001*	0.27
CAL (mm)	Baseline	2.30 ± 0.31	2.34 ± 0.52			
	6 months	1.67 ± 0.53	1.74 ± 0.52	0.60	< 0.001*	0.87
BOP (%)	Baseline	28 ± 26	24 ± 24			
	6 months	11 ± 18	13 ± 13	0.83	< 0.001*	0.47
PI (%)	Baseline	15 ± 28	31 ± 42			
	6 months	6 ± 15	18 ± 26	0.03*	0.10	0.80
RW (mm)	Baseline	3.57 ± 0.60	3.68 ± 0.90			
	6 months	0.93 ± 1.34	1.30 ± 1.23	0.34	< 0.001*	0.57
KTT (mm)	Baseline	1.15 ± 0.24	1.27 ± 0.26			
	6 months	2.00 ± 0.42	2.15 ± 0.57	0.17	< 0.001*	0.83
KTW (mm)	Baseline	2.86 ± 0.91	2.80 ± 0.73			
	6 months	3.63 ± 1.38	3.50 ± 0.79	0.71	< 0.001*	0.82
DHp	Baseline	18.04 ± 22.22	14.71 ± 23.04			
	6 months	9.33 ± 14.60	14.08 ± 22.54	0.89	0.25	0.32
DHe	Baseline	0.15 ± 0.33	0.15 ± 0.40			
	6 months	0.03 ± 0.11	0.06 ± 0.18	0.81	0.04*	0.74
Ep	Baseline	37.25 ± 27.22	33.00 ± 28.07			
	6 months	90.75 ± 13.60	87.00 ± 24.46	0.46	< 0.001*	0.96
RES	6 months	8.47 ± 1.41	7.59 ± 2.73	0.15		
%RC	6 months	84.8 ± 22.3	76.6 ± 22.4	0.213		

*PD* probing depth, *RD* recession depth, *CAL* clinical attachment level, *BOP* bleeding on probing, *PI* plaque index, *RW* recession width, *KTT* keratinized tissue thickness, *KTW* keratinized tissue width, *DHp* dentin hypersensitivity patient, *DHe* dentin hypersensitivity examiner, *Ep* Patient-reported esthetic evaluation, *RES* Root Coverage Esthetic Score, *%RC* percentage of root coverage

Data are presented as mean ± standard deviation

*Statistically significant differences (*p* < 0.05)

No significant correlations were observed between KTT and KTW (baseline and 6 months) and %RC (Table 3).

**Table 3:** Correlation between keratinized tissue parameters and percentage of root coverage (%RC)

Variable	*r*	*p*-value
KTT (baseline)	-0.3075	0.054
KTT (6 months)	-0.2977	0.062
KTW (baseline)	-0.0519	0.750
KTW (6 months)	0.2229	0.167

For the somatosensory analysis, MDT and MPT values were evaluated at baseline, 2 and 6 months. Descriptively, both groups showed slight variations over time, without a consistent pattern of increase or decrease.

For MDT, no significant effects were observed for time (F(2,76) = 0.281, *p* = 0.756), group (F(1,38) = 2.42, *p* = 0.128), or interaction. Similarly, MPT showed no significant differences over time (F(2,76) = 1.46, *p* = 0.238), between groups (F(1,38) = 0.053, *p* = 0.819), or for the interaction (Table 4).

**Table 4:** Somatosensory thresholds (MDT and MPT) at baseline, 2 and 6 months for CHX and PHY groups

Variable	Group	Baseline (median [Q25–Q75])	60 days	6 months	*p*-value (time)	*p*-value (group)	*p*-value (interaction)
MDT	CHX	1.68 (0.39–2.68)	1.60 (0.84–2.15)	1.53 (1.16–1.75)	0.756	0.128	0.753
	PHY	2.08 (0.80–5.20)	1.97 (0.99–3.31)	1.68 (1.08–3.34)			
MPT	CHX	18.3 (9.17–30.3)	19.4 (12.2–40.6)	21.7 (13.1–41.1)	0.238	0.819	0.980
	PHY	29.4 (8.94–38.7)	21.3 (11.5–40.0)	17.7 (12.2–39.5)			

Data are presented as median (interquartile range)

P-values were obtained from two-way repeated measures ANOVA after log10 transformation

**Fig. 5:**
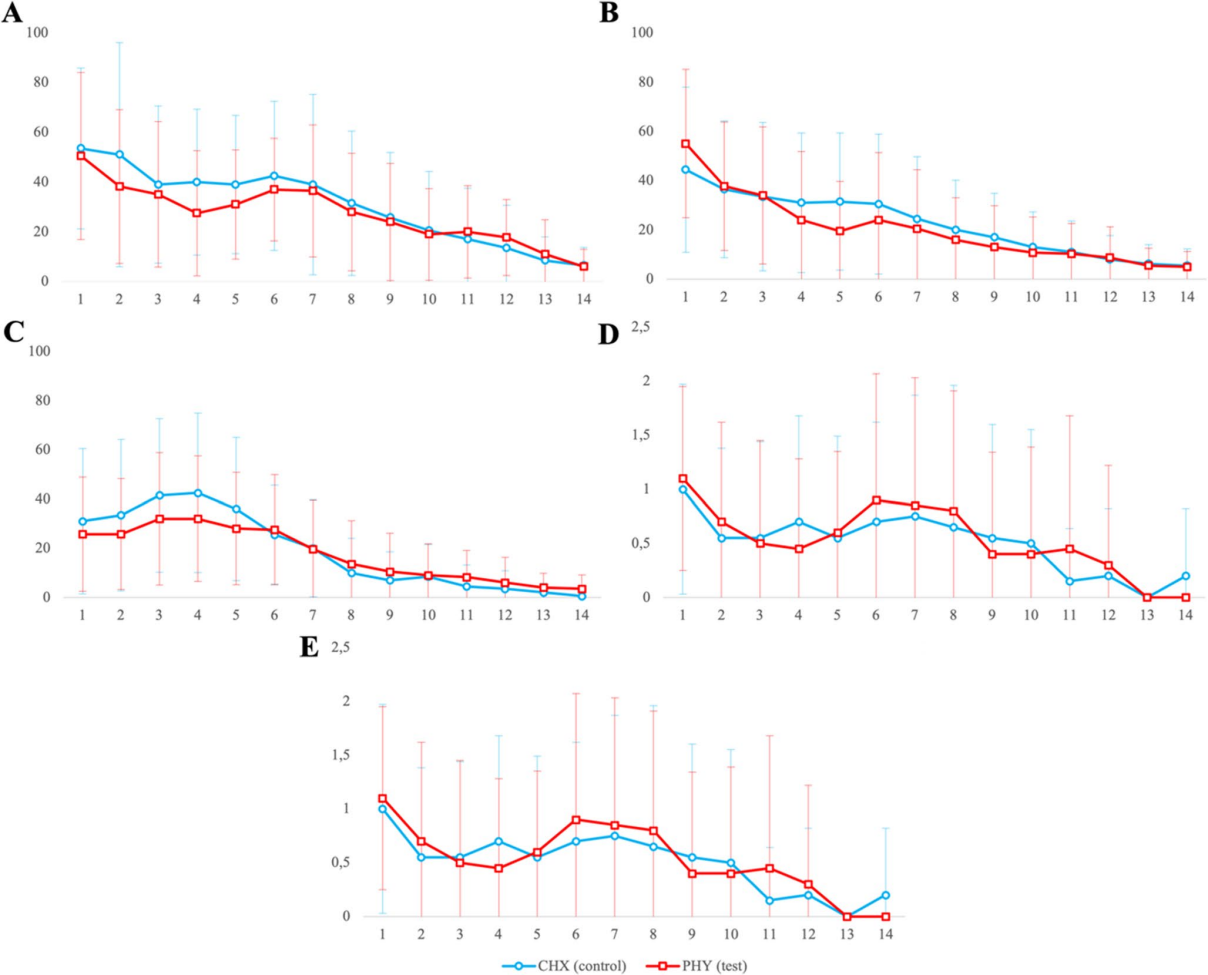
Mean (± standard deviation) patient-reported outcomes over 14 postoperative days for chlorhexidine (CHX, control) and iron phthalocyanine (PHY, test). (**A**) Difficulty in chewing/eating; (**B**) Difficulty in tooth brushing; (**C**) Pain at the graft recipient site; (**D**) Perceived swelling in the mouth/face; (**E**) Analgesic consumption

Patient-reported outcomes assessed using the postoperative diary (Tonetti et al., 2018) showed no differences between groups, but significant improvements over time. Difficulty in chewing decreased progressively from day 3 onward. Difficulty in toothbrushing was highest on day 1 and significantly reduced thereafter. Pain at the recipient sites significantly decreased from day 9, while perceived swelling improved from day 6. Analgesic consumption was highest on day 1 and significantly reduced at subsequent time points (Fig. 5).

No significant differences were observed in OHIP-14 scores between baseline (23 ± 6.84) and 6 months (23.05 ± 9.48) (*p* > 0.05), indicating that oral health-related quality of life remained stable throughout the follow-up period.

## Discussion

The present randomized controlled split-mouth clinical trial demonstrated that the use of an iron phthalocyanine-based mouthrinse during the postoperative period did not compromise the clinical outcomes of root coverage procedures when compared with the conventional 0.12% chlorhexidine protocol. Mean root coverage was 76.6% in the PHY group and 84.8% in the CHX group, with no statistically significant difference between treatments. Likewise, no statistically significant differences were observed between groups regarding the remaining clinical periodontal parameters, professional- and patient-reported outcomes, or the somatosensory profile of the recipient sites. Although the CHX group achieved a numerically higher mean %RC, this difference was not accompanied by differences in CAL, keratinized tissue dimensions, esthetic outcomes, or patient-reported measures. Therefore, under the conditions of the present study, the numerical difference in %RC did not appear to translate into a clinically meaningful difference between the postoperative protocols.

The only difference detected between the groups was a slightly higher PI in the PHY group at 6 months. As no corresponding differences were observed in BOP, CAL, %RC, or any other clinical parameter, this isolated finding is unlikely to be clinically meaningful or to have influenced the healing process or treatment outcomes.

The absence of differences in the primary outcome suggests that PHY did not interfere with graft integration and flap stabilization. Considering that successful root coverage depends on graft revascularization, mechanical stability, and controlled inflammation during early healing [13, 50, 51], these findings indicate that chemical plaque control with PHY was sufficient to maintain favorable conditions for tissue repair.

These results should be interpreted considering previous evidence suggesting potential cytotoxic effects of chlorhexidine on fibroblasts and other cells involved in periodontal healing, including reduced cell proliferation, impaired collagen synthesis, and altered cell migration [21, 52, 53]. In addition, chlorhexidine has been associated with several adverse effects, such as staining and mucosal alterations [23, 24]. In this context, the present findings suggest that iron phthalocyanine demonstrated a favorable postoperative profile during the 6-month observation period, with no evidence of detrimental effects on periodontal wound healing.

From an esthetic perspective, no differences were observed between groups in either professional evaluation using RES or patient perception. The significant improvement in esthetic satisfaction over time in both groups reinforces that the postoperative chemical protocol did not influence the final esthetic outcome. These findings are consistent with previous reports demonstrating the high predictability of CAF combined with SCTG procedures [13, 14, 35, 54, 55].

A strength of the present study is the quantitative assessment of the somatosensory profile of the recipient sites using MDT and MPT. No significant changes were observed between groups during follow-up, suggesting preservation of the functional integrity of the operated areas regardless of the mouthrinse used. These outcomes are clinically relevant given previous reports of transient sensory alterations following periodontal grafting procedures [28, 29, 56]. From a clinical perspective, the present findings demonstrated preservation of recipient-site somatosensory function, providing additional information on potential postoperative sensory alterations, an underexplored aspect of patient experience and surgical sequelae in periodontal plastic procedures.

Patient-centered outcomes followed a similar pattern in both groups, with progressive improvement in pain, chewing ability, oral hygiene performance, and analgesic consumption during the 14-day postoperative period. These results are in agreement with previous clinical studies evaluating postoperative morbidity after mucogingival surgery [57–59]. It is important to note that all participants received the same standardized postoperative pharmacological regimen, in accordance with the ethical requirements and the routine clinical protocol of our institution. Although this regimen may have influenced the overall intensity of postoperative symptoms, its uniform administration to both treatment groups minimized the potential for confounding, ensuring that any observed differences in patient-reported outcomes were unlikely to be attributable to variations in postoperative medication. Beyond the immediate postoperative period, participants also reported a favorable perception of their oral health-related quality of life, as reflected by the low baseline OHIP-14 scores. No clinically meaningful changes were observed at the 6-month follow-up, suggesting that periodontal plastic surgery had a limited impact on overall oral health-related quality of life in this population.

The methodological strengths of this study include the split-mouth design, standardized surgical procedures performed by a single operator, blinded outcome assessment, and the comprehensive evaluation of clinical, esthetic, sensory, and patient-reported outcomes. Although the single-center, single-operator design may limit external validity, it represents a methodological strength by reducing inter-operator variability and improving the consistency and internal validity of the clinical procedures. However, some limitations should be acknowledged. The 6-month follow-up period, although adequate to assess early healing and short-term stability, does not allow conclusions regarding long-term outcomes. Additionally, due to the sequential nature of the interventions, possible carry-over effects represent a limitation inherent to this study design. Furthermore, although the sample size was adequately calculated for the primary outcome, it may have limited the ability to detect subtle differences in secondary outcomes. Nevertheless, these secondary outcomes, mainly somatosensory evaluation, provided valuable preliminary clinical information and may contribute to the design of future studies. In addition, the inclusion of both RT1 and RT2 GRs may have introduced some degree of clinical heterogeneity. However, this approach reflects the variety of recession defects commonly encountered in clinical practice. Moreover, the split-mouth design allowed direct within-patient comparisons, as contralateral defects were carefully selected to present similar baseline characteristics, thereby minimizing the potential influence of this variability on treatment outcomes.

From a clinical perspective, the present findings suggest that iron phthalocyanine may be used as a postoperative antiseptic following periodontal plastic surgery without compromising clinical, esthetic, somatosensory, or patient-reported outcomes compared with chlorhexidine. Given the increasing interest in minimizing the adverse effects of chlorhexidine and improving biocompatibility, these results contribute to expanding evidence-based therapeutic options.

## Conclusions

Within the limitations of this split-mouth clinical trial, postoperative use of iron phthalocyanine following CAF plus SCTG presented favorable clinical, esthetic, sensory, and patient-reported outcomes, with no statistically significant differences compared with chlorhexidine during the 6-month follow-up period. Further randomized clinical trials with larger sample sizes and longer follow-up are warranted to confirm these findings and to evaluate long-term periodontal stability.

## Data Availability

The datasets generated and/or analyzed during the current study are not publicly available due to privacy and ethical restrictions but are available on reasonable request.
